# Elevated Antibacterial Activity of a Polygalacturonic + Caprylic Acids Wound Ointment Compared with Hypochlorous Acid in a Three-Dimensional Wound Biofilm Model

**DOI:** 10.3390/pathogens15020188

**Published:** 2026-02-08

**Authors:** Bahgat Gerges, Joel Rosenblatt, Y-Lan Truong, Ying Jiang, Issam Raad

**Affiliations:** Department of Infectious Diseases, Infection Control and Employee Health, MD Anderson Cancer Center, The University of Texas, Houston, TX 77030, USA; jsrosenblatt@mdanderson.org (J.R.); yltruong@mdanderson.org (Y.-L.T.); yijiang@mdanderson.org (Y.J.); iraad@mdanderson.org (I.R.)

**Keywords:** polygalacturonic + caprylic acids (PG + CAP), hypochlorous acid (HOCl), fibrin-gel wound biofilm (FGWB) model, methicillin resistant *Staphylococcus aureus* (MRSA), multi-drug resistant (MDR) *P. aeruginosa*, metallo β-Lactamase *E. coli*, *Streptococcus pyogenes*

## Abstract

Bacterial biofilms play a major role in delayed wound-healing and in the development of chronic, non-healing wounds. Natural, plant-based agents, which can eradicate bacterial biofilms, are alternatives to antibiotics and antiseptics in the treatment of bacterially contaminated wounds. Bacterial wound biofilms are three-dimensional and complex microbial communities. Therefore, in this study, we used a three-dimensional fibrin-gel wound biofilm (FGWB) model to compare a commonly used natural agent in wound care, hypochlorous acid (HOCl), to a combination of two natural plant-based agents, polygalacturonic acid (PG) and caprylic acid (CAP) (PG + CAP), for their abilities to eradicate resistant bacterial biofilms of common wound pathogens methicillin resistant *Staphylococcus aureus* (MRSA), multi-drug resistant (MDR) *Pseudomonas aeruginosa*, metallo β-Lactamase *Escherichia coli*, and *Streptococcus pyogenes*. PG + CAP produced a significantly greater reduction in viable organisms when compared to HOCL (*p* ≤ 0.05) against all tested bacterial isolates. PG + CAP was highly effective against biofilms of all resistant bacterial isolates and is a promising option that merits further study for treating chronic wounds contaminated with bacterial biofilms.

## 1. Introduction

Bacterial biofilms play a significant role in generating prolonged inflammation that delays healing and produces chronic wounds [1]. Treating bacterial chronic-wound biofilms therefore is a significant problem and is one of the main reasons for the development of resistant bacterial isolates when antibiotic ointments are used [2,3]. In contrast to antibiotics, antiseptics, which can be bactericidal or bacteriostatic, are used to reduce the presence of bacteria in wounds without inducing antibiotic-resistant bacteria [3,4,5]. However, the use of antiseptics during wound-healing has been associated with cytotoxicity [6,7]. Cytotoxicity also produces inflammation in wounds and contributes to delayed wound-healing by degrading newly deposited granulation tissues [7,8,9,10]. Natural agents, which can eradicate bacterial biofilms without leading to antibiotic resistance or cytotoxicity, are alternatives to antiseptics in the treatment of bacterially contaminated wounds that potentially offer an improved balance of antimicrobial activity and wound-healing [11].

Biofilm-producing bacteria are of great importance from a public-health perspective because they cause 80% of the infections that occur in the human body [12]. Chronic wounds represent a significant economic burden, including $25 billion dollars annually in the United States alone [12,13,14]. Bacterial biofilms comprise microbial communities surrounded by extracellular matrices of polymeric macromolecules [3]. Bacteria within the biofilm become less susceptible to antimicrobial agents through physical barriers to access created by the biofilm extracellular matrix as well as by phenotypic adaptations including altering metabolism, genetic regulation, and protein expression [3,15]. Many traditional biofilm models have been used to study the efficacy of antimicrobial agents against pathogenic bacterial biofilms [16,17,18], but these traditional models are not representative of the three-dimensional matrix of microbes found in wound biofilms [3,16]. Therefore, in the current study, we used a three-dimensional fibrin-gel wound biofilm (FGWB) model to be more representative of the three-dimensional extracellular matrix in which bacterial wound biofilms are actually enmeshed.

Here, the authors report studies of the comparative efficacies of a novel combination of two natural plant-based agents, polygalacturonic acid (PG) and caprylic acid (CAP), versus the widely used natural wound-disinfectant, hypochlorous acid (HOCl), in both solution- and gel formulations for eradicating bacterial biofilms using a three-dimensional FGWB model. Previous studies have shown that PG + CAP is highly effective against pathogenic bacterial isolates in biofilms both in vitro using a traditional two-dimensional biofilm model as well as in vivo [19,20]. Preliminary data demonstrating the safe application of PG and CAP in combination to human chronic wounds was reported in a pilot human clinical trial [21]. In an acute porcine full-thickness wound study, the combination of PG and CAP was well-tolerated over multiple daily applications and did not impede normal tissue healing [21]. The mechanism of action of PG + CAP against microbial pathogens derives from the synergy of the medium-chain fatty acid caprylic acid (CAP), which disrupts the cell-membrane integrity [22,23,24], while polygalacturonic acid (PG) plays an important role in maintaining an optimal CAP bactericidal pH and for enhancing the bioavailability of low-solubility CAP by emulsifying it into microdroplets to help it to penetrate the biofilm and exert its bactericidal action on resident cells [11,25].

HOCl in solution and gel forms are commercially available antimicrobial wound treatments and have demonstrated rapid inhibition of bacteria in wound beds through disruptive reactions with different bacterial lipids, nucleotides, and proteins [26,27]. Although the mechanism of action of HOCl against microorganisms is not completely understood, many studies have reported that HOCl rapidly kills microbes through these oxidative chemical reactions [28,29,30,31,32,33].

## 2. Materials and Methods

### 2.1. Antibacterial Agents

The authors compared the efficacy of a novel PG + CAP ointment with that of HOCl solution and HOCl gel (PG: Sigma-Aldrich Inc., St. Louis, MO, USA, catalog #P3889-100G, CAS #25990-10-7; CAP: Sigma-Aldrich, catalog #03907, CAS #124-07-2; HOCl solution: Aqua Science Inc., Columbus, OH, USA, catalog #01S.06E, CAS #7790-92-3; HOCl gel: active skin repair hydrogel, lot #HG016F25, Part #14001B v.4.0). PG + CAP ointment was prepared in a laboratory as previously described by Gerges et al. 2021 [19], and the HOCl wound solution and HOCl gel formulations were used as directed by the manufacturers. An aqueous gel containing 2-hydroxyethylcellulose (Sigma-Aldrich Inc., St. Louis, MO, USA, catalog #434973-250G. CAS #9004-62-0. Steinheim, Germany.) was used as the ointment base for PG + CAP ointment.

### 2.2. Three-Dimensional FGWB Model

A quantitative, in vitro, three-dimensional FGWB model adapted from the model of Besser and Stuermer 2019 [34] and prepared according to the specifications of Truong et al. 2022 [35] and Gerges et al. 2025 [36] was used in the current study. Briefly, 20 mg/mL fibrinogen (Fisher Scientific, catalog #34-157-61GM) was slowly dissolved in phosphate-buffered saline (PBS) at 37 °C; 5 units/mL thrombin (Sigma-Aldrich, catalog #T7009-100UN; CAS #9002-04-4) was dissolved in PBS; and 125 mM calcium chloride (Sigma-Aldrich, catalog #C3306-100G, CAS #10035-04-8) was dissolved in deionized water and added to initiate gelation, and the fibrin-gel wound biofilm (FGWB) was formed by adding 1.5 mL of the above combination in 24-well tissue culture plate and left in the refrigerator until being used as described by Truong et al. 2022 [35], and Gerges et al. 2025 [36]. Briefly, a total of 2 mL of human plasma containing 10^4^ colony-forming units per milliliter (CFU/mL) of each tested bacterial isolate was added to the previously prepared 1.5 mL of the FGWB gel model and then incubated for 48 h at 37 °C, forming disk-shaped biofilms. Forty-eight hours was selected as the incubation duration to ensure that mature biofilms had formed. All culture liquids were then removed, and the colonized fibrin-gel disks were washed for 30 min in isotonic sterile saline to remove any remaining planktonic organisms.

### 2.3. Biofilm Eradication Assay

Biofilm eradication testing was conducted using highly virulent clinical bacterial isolates of MRSA (MDA #120), multi-drug-resistant (MDR) *Pseudomonas aeruginosa* (MDA #118), metallo β-Lactamase carbapenem-resistant Enterobacterales (CRE) *Escherichia coli* (MB #9245), and *Streptococcus pyogenes* (MB #3175) as representative hospital-acquired infection pathogens from cancer patients. The organisms were grown from glycerol stock on trypticase soy agar + 5% sheep blood (Remel, Lenexa, KS, USA, reference #R01202). Each organism was inoculated into Muller Hinton broth (Fisher Scientific, BBL Mueller Hinton II Broth Cation Adjusted, Sparks, MD, USA, catalog #B12322) and diluted to 0.5 McFarland. Additional dilutions were made to obtain ~10^4^ colony-forming units/mL (CFUs/mL). The efficacy of PG + CAP versus HOCl was determined using the FGWB model as described by Gerges et al. 2025 [36]. Twelve technical replicates of FGWB were used for each of PG + CAP and negative control, and six technical replicates were used for each of the HOCl solution- and gel forms against each organism. Regrowth experiments were conducted, as described by Gerges et al. 2021 and 2025 [19,36], to ensure that eradication was complete on FGWB disks from which no viable colonies were recovered following exposure to antimicrobial agents.

### 2.4. Statistical Analysis

Colony-forming unit/milliliter (CFU/mL) values for each bacterial isolate were evaluated across groups. The PG + CAP group was compared with the HOCl solution and HOCl gel groups using the Wilcoxon rank-sum test. Log_10_ reductions in median CFU relative to the negative control group were also assessed for each treatment group. All tests were two-sided, with a significance level of ≤0.05. Data analyses were performed using SAS version 9.4 (SAS Institute Inc., Cary, NC, USA).

## 3. Results

**Figure 1:**
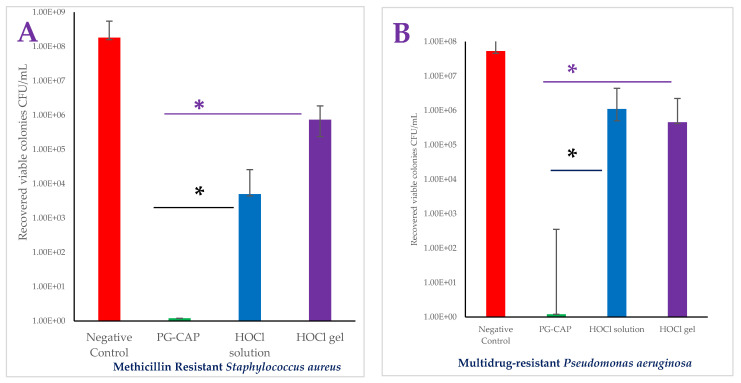

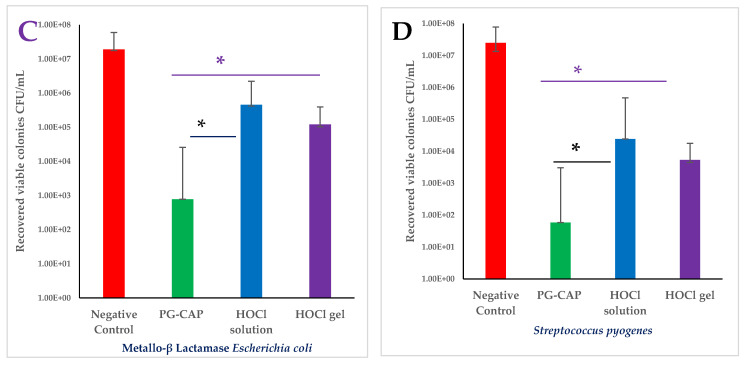
(**A**–**D**): Efficacies of PG + CAP, HOCl solution, and HOCl gel against tested bacterial biofilms after three hours exposure using fibrin-gel wound biofilm model against MRSA (**A**), *Pseudomonas aeruginosa* (**B**), Metallo-β Lactamase *Escherichia coli* (**C**), and *Streptococcus pyogenes* (**D**). Values are presented as the median number of recovered viable colony-forming units/mL (CFUs/mL) for twelve technical replicates for PG + CAP and control, and six technical replicates for HOCl solution and HOCl gel. Bars indicate the median numbers of recovered viable colonies, and error bars indicate the interquartile ranges. (*) Indicates statistically significant differences between PG + CAP and each HOCl solution and HOCl gel (*p* ≤ 0.05).

**Figure 2:**
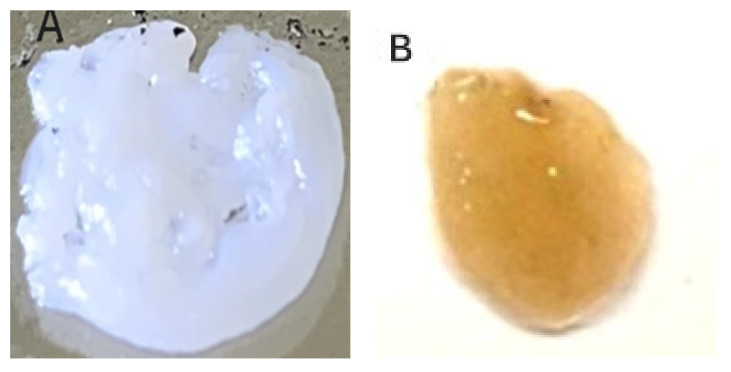

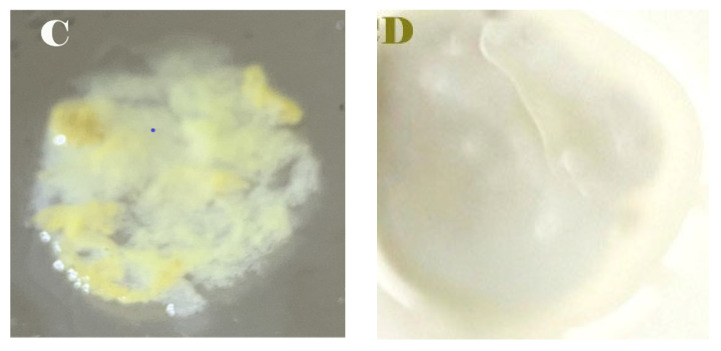
Shows the fibrin-gel wound biofilm model (**A**) before (fibrin-gel control) and (**B**) after MRSA inoculation and biofilm formation (MRSA biofilm control). (**C**,**D**) are the fibrin-gel wound biofilm after 3 h of exposure to HOCl gel (**C**) and PG + CAP (**D**). The gold-yellow color in (**B**,**C**) indicate viable MRSA organisms that are present in the fibrin-gel matrices. The white color in (**A**,**D**) are due to fibrin, and the absence of gold-yellow tint indicates that there are no viable MRSA organisms in those fibrin-gel matrices.

**Figure 3:**
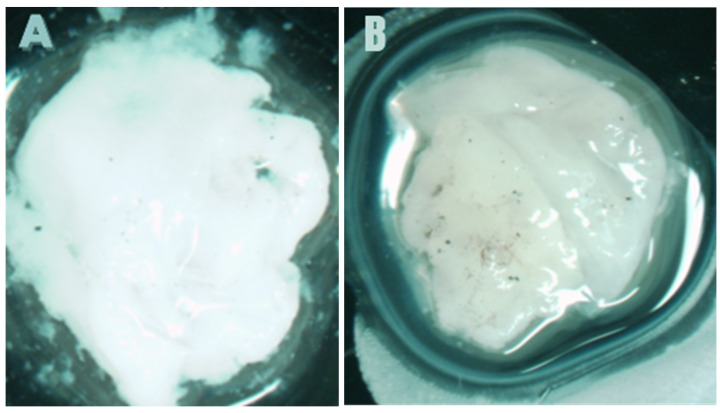

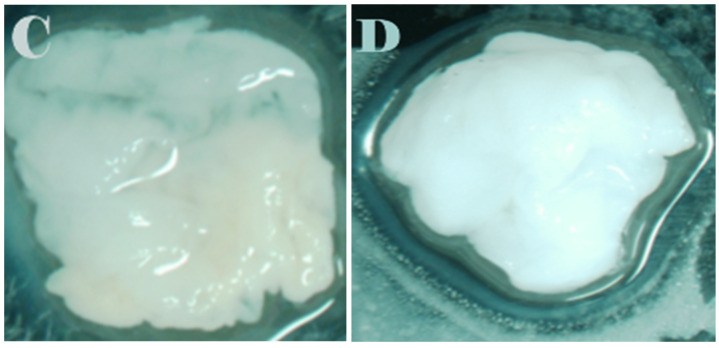
Shows the fibrin-gel wound biofilm model (**A**) before (fibrin-gel control) and (**B**) after Pseudomonas aeruginosa (PA) inoculation and biofilm formation (PA biofilm control). (**C**,**D**) are the fibrin-gel wound biofilm after 3 h of exposure to HOCl gel (**C**) and PG + CAP (**D**). The greenish tint in (**B**,**C**) indicate viable PA organisms that are present in the fibrin-gel matrices. The white color in (**A**,**D**) are due to fibrin, and the absence of green tint indicates that there are no viable PA organisms in those fibrin-gel matrices.

Figure 1 presents the median recovered viable colonies (CFUs/mL) of tested infectious bacterial biofilms of MRSA (Figure 1A), MDR *P. aeruginosa* (Figure 1B), metallo β-Lactamase carbapenem-resistant Enterobacterales (CRE) *Escherichia coli* (Figure 1C), and *St. pyogenes* (Figure 1D) after three hours of exposure to PG + CAP, HOCl solution, or HOCl gel in a FGWB model. PG + CAP reduced viable MRSA CFUs/mL by 8.26 log_10_, HOCl solution, and gel reduced MRSA CFUs/mL by 4.56 log_10_, and 2.39 log_10_, respectively. PG + CAP, HOCl solution, and HOCl gel reduced viable MDR *P. aeruginosa* CFUs/mL by 7.56 log_10_, 1.68 log_10_, and 2.06 log_10_, respectively, metallo β-Lactamase CRE *E. coli* CFUs/mL by 4.38 log_10_, 1.68 log_10_, and 2.20 log_10_, respectively, and *S. pyogenes* by 5.63 log_10_, 3.01 log_10_, and 3.67 log_10_, respectively. The antibacterial superiority of PG + CAP relative to HOCl was statistically significant (*p* ≤ 0.05) against all tested bacterial isolates. Figure 2 shows fibrin-gel wound biofilm before (A) and after MRSA biofilm formation (B), while (C) and (D) present FGWB after three hours of exposure to PG + CAP and HOCl gel, respectively. Figure 3 shows fibrin-gel wound biofilm before (A) and after (B) MDR *Pseudomonas aeruginosa* biofilm formation, while (C) and (D) present FGWB after three hours of exposure to PG + CAP and HOCl gel, respectively.

## 4. Discussion

Acute inflammation plays an important role in normal wound-healing. The acute response to dermal wounds includes recruitment of neutrophils, macrophages, and lymphocytes which clear damaged tissue and colonizing bacteria through phagocytosis, secretion of reactive oxidative moieties (such as HOCl) and secretion of proteases [37,38]. Subsequently, a key part of normal wound-healing as inflammation subsides is the transition to the proliferation and remodeling phases in which cytokine-recruited fibroblasts and keratinocytes take over key roles [39]. Fibroblasts play key roles in generating new granulation tissue over which keratinocytes can proliferate to re-epithelialize the wound [40].

Chronic non-healing wounds occur when the inflammatory phase becomes entrenched, preventing transition to the proliferation phase [41]. Aberrant inflammation associated with chronic non-healing wounds frequently contains bacteria that are able to survive and evade immune reactions or antibiotics by forming protective biofilms [42]. Hence, therapies to restore normal healing of chronic wounds need to be able to eradicate bacteria in wound biofilms. In the current study, we allowed bacteria to form mature protective biofilms enmeshing in both bacterial extracellular matrix as well as fibrin, an abundant protein present in inflammatory wound exudate [43]. Our experiments showed a single treatment of PG + CAP for three hours was highly effective in eradicating bacteria in the mature fibrin-gel biofilms but did not fully eradicate all bacteria. MRSA and MDR *P. aeruginosa* biofilms were fully eradicated; however, metallo β-Lactamase *E. coli* and *S. Pyogenes* had reduced viable bacterial concentrations (over 4 Log_10_ reductions) but were not fully eradicated in a single treatment. The three hours of exposure was selected as a representative duration over which the applied ointment would not be expected to become excessively diluted by diffusion from the wound bed or dilution by wound exudate. The tested bacteria are common, resistant dermal-wound pathogens associated with chronic non-healing wounds [44,45]. The PG + CAP results suggest multiple continued applications of PG + CAP would be required to sustain minimal bacterial bioburden in chronic wounds allowing granulation tissue formation to proceed without excessive inflammatory disruption. HOCl was significantly less effective than PG + CAP in reducing bacterial bioburden in fibrin-gel biofilms against all tested bacteria. This suggests once biofilm matures, a robust bacterial population could survive HOCl irrigation and continue inducing inflammatory responses.

HOCl exerts its antibacterial activity primarily by oxidative chemical reactions [46]. It may therefore be limited in the three-dimensional fibrin-gel wound biofilms by being consumed through non-specific oxidative reactions with extracellular matrix molecules. Bacterial extracellular matrix and fibrin are rich in oxidizable primary and side chains that can rapidly consume the HOCl before they are able to react with resident bacteria [47]. Our finding of limited HOCl antibacterial efficacy against bacteria embedded in complex matrices like wound biofilms has similarly been reported by other authors [45,48]. PG + CAP synergistically works by CAP disrupting bacterial membranes with PG providing optimal pH and emulsifying CAP for optimal bioavailability [11,49]. CAP has been shown to provide an optimal lipid chain length for selectively minimizing disruption of human cell-membranes while killing bacteria [49]. Longer chain fatty acids produce progressively increased human cell-membrane disruption [49,50]. In addition, PG has been shown to inhibit destructive wound matrix metalloproteases (MMP-2 and MMP-9) produced during inflammation responses while maintaining wound hydration [51]. PG is derived from pectin in tree fruits, such as apple, and is readily available at low cost [52]. CAP can be extracted from coconut and is also readily available at low cost [53,54]. Thus, PG + CAP is expected to be a practical plant-based wound-treatment technology.

Our study has several limitations. It was entirely in vitro; thus, in vivo verification is required. A pilot six-week human clinical trial demonstrated the safety and effectiveness of PG + CAP in improving chronic wounds in humans [20], and an acute porcine wound study demonstrated PG + CAP ointment was well-tolerated [21], but additional in vivo study is required. The models used in this study attempted to replicate many real complexities of chronic-wound biofilm extracellular matrixes; however, the full complexity of biofilms in wound beds may not have been entirely replicated. Also, wounds frequently are colonized by multiple microbial species, while our models were single species. Since optimal wound-healing requires a balance of potent antimicrobial activity with minimal cytotoxicity towards fibroblasts, keratinocytes and other immune cells, additional cytotoxicity studies on PG + CAP are warranted. Nevertheless, our results showed that HOCl may have limited effectiveness in chronic wounds heavily colonized with mature bacterial biofilms and that PG + CAP is a promising alternative with superior antibacterial biofilm properties that merits further in vitro and in vivo study.

## Data Availability

The original contributions presented in this study are included in the article. Further inquiries can be directed to the corresponding author.

## Abbreviations

The following abbreviations are used in this manuscript:

FGWB	Fibrin-gel wound biofilm
HOCl	Hypochlorous acid
PG	Polygalacturonic acid
CAP	Caprylic acid
PG + CAP	Polygalacturonic acid + caprylic acid
MRSA	Methicillin resistant *Staphylococcus aureus*
MDR *P. aeruginosa*	Multi-drug resistant *Pseudomonas aeruginosa*
CRE	Carbapenem-resistant Enterobacterales
